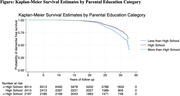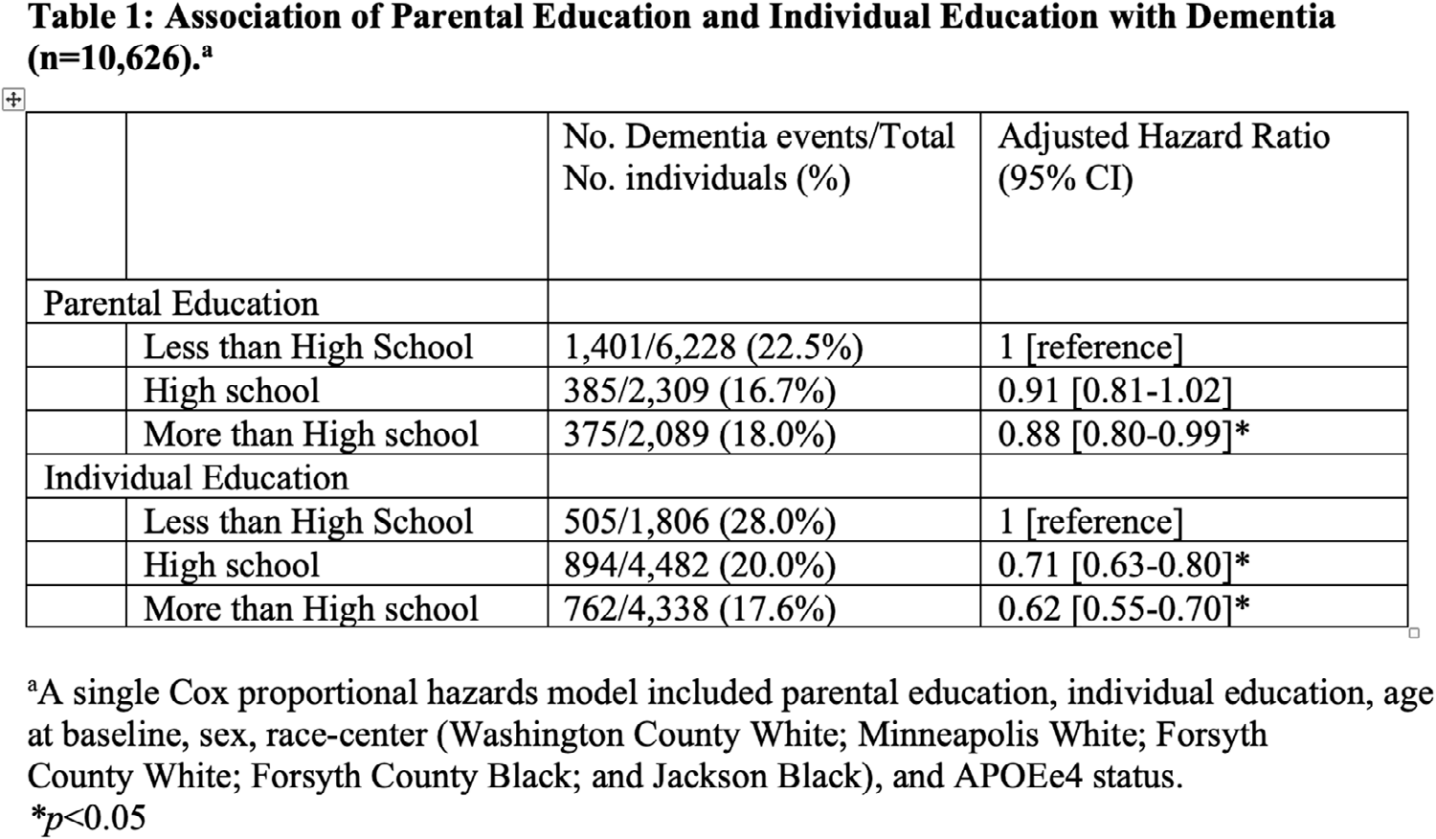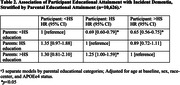# Intergenerational educational attainment and risk for incident dementia: The Atherosclerosis Risk in Communities [ARIC] Neurocognitive Study

**DOI:** 10.1002/alz.091858

**Published:** 2025-01-09

**Authors:** Soumya Gupta, Valerie Morrill, Renée C. Groechel, Anna M. Kucharska‐Newton, Thomas H. Mosley, Pamela L. Lutsey, Erika Meza, Rebecca F. Gottesman

**Affiliations:** ^1^ The Johns Hopkins University School of Medicine, Baltimore, MD USA; ^2^ National Institute of Neurological Disorders & Stroke Intramural Research Program, National Institute of Health, Bethesda, MD USA; ^3^ University of North Carolina Gillings School of Global Public Health, Chapel Hill, NC USA; ^4^ University of Mississippi Medical Center, Jackson, MS USA; ^5^ University of Minnesota School of Public Health, Minneapolis, MN USA; ^6^ Harvard University, Cambridge, MA USA

## Abstract

**Background:**

Individuals with higher educational attainment have a lower risk for dementia, but it is less clear how educational attainment of an individual’s parents may influence dementia risk, particularly in diverse populations. The study goal was to investigate the association between intergenerational educational attainment for two adjacent generations (individuals and their parents) and the risk of incident dementia, in a community‐based study.

**Method:**

Participants from the Atherosclerosis Risk in Communities (ARIC) study, a prospective community‐based cohort, were asked their own educational level (at study baseline; 1987‐1989; ages 44‐66 years) and that of each of their parents several years later. Dementia status was ascertained from baseline through 2020 using expert committee diagnoses from comprehensive neuropsychological assessments, informant interviews, telephonic assessments, hospitalization codes, and death certificates. Participants without dementia at baseline, and with non‐missing covariates and educational history were included. Educational level was classified (for participants and the higher‐educated parent) as less than high school (<HS); high school or GED (HS); and more than high school (>HS). We defined “overachievement” as a participant having higher educational attainment than their parent. Cox‐proportional hazard models were used to evaluate the association between intergenerational educational attainment and incident dementia risk.

**Result:**

Among 10,626 participants (21.8% Black; 56.0% female) the overall dementia incidence rate was 7.9 [95% CI, 7.6‐8.3] per 1000 person‐years, with differences by educational category (Figure 1; log‐rank p<0.0001). High parental education level (>HS: HR, 0.88; 95% CI, 0.80‐0.99) and individual education level (>HS: HR, 0.62; 95% CI, 0.55‐0.70) were each significantly and independently associated with a lower risk of dementia (Table 1). Additionally, among participants whose parents had <HS education, overachieving parental education (i.e. participant has completed HS (HR, 0.69; 95% CI, 0.60‐0.79) or completed >HS (HR, 0.65; 95% CI, 0.56‐0.75)), as compared to matching parental education, was significantly associated with lower dementia risk (Table 2).

**Conclusion:**

Individuals with parents of higher educational attainment exhibit a reduced risk of dementia, and higher individual educational level is associated with reduced dementia risk, independent of parental education. Moreover, surpassing parental educational attainment, rather than matching it, is associated with a reduced risk of dementia.